# IL-1RAcPb signaling regulates adaptive mechanisms in neurons that promote their long-term survival following excitotoxic insults

**DOI:** 10.3389/fncel.2013.00009

**Published:** 2013-02-15

**Authors:** David Gosselin, Marc-André Bellavance, Serge Rivest

**Affiliations:** Faculty of Medicine, Neuroscience Laboratory, CHU de Québec Research Center and Department of Molecular Medicine, Laval UniversityQuebec City, QC, Canada

**Keywords:** interleukin-1, IL-1RAcPb, excitotoxicity, neuronal death, kainic acid, calcium, calpastatin, brain-derived neurotrophic factor

## Abstract

Excitotoxicity is a major component of neurodegenerative diseases and is typically accompanied by an inflammatory response. Cytokines IL-1alpha and IL-1beta are key regulators of this inflammatory response and modulate the activity of numerous cell types, including neurons. IL-1RAcPb is an isoform of IL-1RAcP expressed specifically in neurons and promotes their survival during acute inflammation. Here, we investigated *in vivo* whether IL-1RAcPb also promotes neuronal survival in a model of excitotoxicity. Intrastriatal injection of kainic acid (KA) in mice caused a strong induction of IL-1 cytokines mRNA in the brain. The stress response of cortical neurons at 12 h post-injection, as measured by expression of Atf3, FoxO3a, and Bdnf mRNAs, was similar in WT and AcPb-deficient mice. Importantly however, a delayed upregulation in the transcription of calpastatin was significantly higher in WT than in AcPb-deficient mice. Finally, although absence of AcPb signaling had no effect on damage to neurons in the cortex at early time points, it significantly impaired their long-term survival. These data suggest that in a context of excitotoxicity, stimulation of IL-1RAcPb signaling may promote the activity of a key neuroprotective mechanism.

## Introduction

Neuronal dysfunctions and damage associated with excitotoxic insults are intrinsically linked with aberrant calcium signaling in neurons and are implicated in a wide variety of neurodegenerative diseases, including strokes, epilepsy and Alzheimer's disease. Shortly after the initiation of these insults, an inflammatory response driven by microglia and astrocytes is triggered to promote the re-establishment of neuronal homeostasis and functions. Members of the interleukin 1 family of cytokines, including IL-1α and IL-1β, are among the most important and pleiotropic regulators of the inflammatory response associated with excitotoxic insults. Binding of either cytokines to the IL-1 receptor type 1 (IL-1R1) causes its heterodimerization with IL-1 receptor accessory protein (IL-1RAcP, or AcP) (Korherr et al., 1997; Cullinan et al., 1998). This complex then recruits adaptor protein MyD88, which then allows for the activation of signaling pathways that regulate the activity of MAP kinases and NF-κB transcription factors (Muzio et al., 1997; Medzhitov et al., 1998).

A strong body of evidence suggests that IL-1 signaling enhances neuronal damage in a context of excitotoxicity (Relton and Rothwell, 1992; Vezzani et al., 2000; Viviani et al., 2003; Bender and Baram, 2007; Andoh et al., 2008; Kwon et al., 2010; Zheng et al., 2010; Maroso et al., 2011). Indeed, IL-1β potentiated NMDA-induced increase in intracellular calcium and led to an exacerbation of neuronal cell death *in vitro* (Viviani et al., 2003). In mice, inhibition of IL-1β by intrastriatal injection of recombinant IL-1 receptor antagonist (IL-1RA), an endogenous antagonist of IL-1β, protected neurons from the excitotoxic effects of a NMDA receptor agonist injection (Relton and Rothwell, 1992). Similar effects were noted in transgenic mice engineered to overexpress high levels of the human IL-RA in the brain (Vezzani et al., 2000). Finally, IL-1β synthesis also correlated with increased neuronal death and increased seizure activity (Kwon et al., 2010; Maroso et al., 2011). Therefore, with respect to excitotoxic insults, IL-1 signaling appears to increase neuronal dysfunctions and death.

In contrast, others have reported a protective role for IL-1 cytokines during excitotoxicity (Ohtsuki et al., 1996; Carlson et al., 1999; Bernardino et al., 2005; Durukan and Tatlisumak, 2010; Hayakawa et al., 2010; Wang et al., 2010; Mayado et al., 2011). For instance, IL-1α and IL-1β both increased survival of neurons stimulated by prolonged exposure to a NMDA agonist by promoting expression of nerve growth factor (Carlson et al., 1999). Furthermore, other studies indicated that IL-R1 signaling is critical to establish the protective effects of tolerance induced by a variety of preconditioning regimen on excitotoxicty-associated neuronal damage (Ohtsuki et al., 1996; Durukan and Tatlisumak, 2010; Mayado et al., 2011). Finally, the role of IL-1 cytokines could be bi-phasic, with dosage and timing parameters being critically implicated (Bernardino et al., 2005; Hayakawa et al., 2010). Indeed, whereas pre-incubation of organotypic hippocampal slices with a relatively low dose of recombinant IL-1β enhanced AMPA-induced neuronal toxicity, a higher dose of IL-1β protected neurons (Bernardino et al., 2005). In light of these observations, the overall impact of IL-1 cytokines in mediating neuronal survival or death appears to be context dependent. Whereas a relatively elevated level of IL-1 signaling before the initiation of an excitotoxic insult stimulates the induction of tolerance in neurons, post-insult signaling seems to favor a detrimental outcome on neuronal homeostasis.

Interestingly, a recent study suggested that post-lesion IL-1 signaling mediated by IL-1RAcPb (AcPb) could provide significant neuroprotection (Smith et al., 2009). AcPb is an isoform of AcP derived from an alternative splicing of exon 12 in the C-terminal that is only 35% similar to that of AcP, and yields a mature protein that possesses 140 additional amino acids in its C-terminal (Smith et al., 2009). Interestingly, AcPb expression is restricted to neurons. Though both proteins modulate MAP kinases activity and in particular the p38 pathway, AcPb, unlike AcP, does not activate canonical NF-κB transcription factors (Huang et al., 2011; Nguyen et al., 2011). Consequently, AcPb signaling has relatively marginal effects of gene transcription compared to AcP. However, AcPb activity enhances calcium influx following N-Methyl-D-aspartic acid (NMDA)-induced stimulations by modulating Src phosphorylation (Huang et al., 2011). Thus, it appears to tune synaptic and neuronal activities. Interestingly, mice deficient for AcPb exhibited more neuronal damage than WT mice following an intracerebral injection of the potent pro-inflammatory toll-like receptor 4 ligand lipopolysaccharide (LPS) (Smith et al., 2009).

These latter properties of AcPb are quite interesting as they suggest that AcPb could modulate calcium signaling and thus regulate the ability of neurons to cope with aberrant calcium regulations, as they occur for example with excitotoxic insults. Therefore, the present study tested the hypothesis that AcPb signaling modulates neuronal survival during excitotoxic insults induced by an injection of kainic acid (KA). KA is a potent agonist of the ionotropic glutaminergic receptors Kainate and AMPA (Wang et al., 2005). Intracerebral injection of KA causes excessive neuronal influx of Ca^2+^, oxidative stress, and mitochondrial dysfunctions leading to neuronal death through multiple mechanisms of necrosis and apoptosis (Zheng et al., 2011). Using mice deficient for AcPb, we demonstrate that AcPb signaling confers cortical neurons significant long-term protection against the excitototoxic effects of an intracerebral injection of KA.

## Materials and methods

### Animals

Adult male C57BL/6J mice were purchased at 7–8 weeks of age from Taconic. Male IL-1RAcPb-deficient mice (AcPb^−/−^), on a C57Bl/6J background, were generated as previously described and then bred in-house (Smith et al., 2009). All animals (25–30 g) were acclimated to standard laboratory conditions (14 h light, 10 h dark cycle; lights on at 06:00 and off at 20:00 h) with free access to rodent chow and water. All protocols were conducted according to the Canadian Council on Animal Care guidelines, as administered by the Laval University Animal Welfare Committee.

### Kainic acid stereotaxic injection

Mice were anesthetized with isofluorane, and the site of injection was stereotaxically reached (David Kopf Instruments, Tujunga, CA). The coordinates from bregma were 0.0 mm anterior-posterior, −2.0 mm lateral and −3.2 mm dorsoventral in order to reach the right striatum, as identified in the mouse brain atlas Paxinos and Franklin (2001). A volume of 2.0 μl containing 150 ng of KA (Sigma) diluted in vehicle (sterile 0.9% saline) was infused over 2 min by means of a 28-gauge stainless steel cannula (Plastics One, Roanoke, VA) that was connected to a 50 μl Hamilton syringe mounted on a UltraMicroPump II controlled by microprocessor controller Micro4 (World Precision Instruments). Control mice were injected with 2.0 μl of vehicle. A 2 min rest was allowed after the injections, and the syringe was then raised incrementally over a period of 2 min. Mice received 1.0 ml of saline subcutaneously after the injection, and were housed one per cage until sacrifice.

### Perfusion and tissue preparation

Mice were anesthetized at different time points (12 h, 48 h, and 15 days post injection) with an intraperitoneal injection (100 μl) of ketamine hydrochloride (91 mg/ml) and xylazine (9.1 mg/ml) and then rapidly perfused transcardially with 0.9% saline, followed by 4% paraformaldehyde (PFA) either in 0.1 M borax buffer, pH 9.0 at 4°C. After perfusion, brains were rapidly removed from the skulls, postfixed for 4 days, and then placed in a solution containing 20% sucrose diluted in 4% PFA/3.8% borax buffer (pH 9.0). The frozen brains were mounted on a microtome (Reichert–Jung, Cambridge Instruments, Deerfield, IL), frozen with dry ice, and cut into 25-μm coronal sections from the start of the prefrontal cortex to the end of the cerebral cortex. The slices were collected in a cold cryoprotective solution (0.05 M sodium phosphate buffer, pH 7.3, 30% ethylene glycol, 20% glycerol) and stored at −20°C.

### *In situ* hybridization (ISH) and immunohistochemistry (IHC)

Standard ISH was performed on every 12th coronal section of the entire rostro-caudal extent of each brain using ^35^S-labeled cRNA probes as described previously (Laflamme and Rivest, 1999; Laflamme et al., 2003). See Table 1 for the list of cRNA probes used. Some sections were processed for double-label IHC and ISH, using an antibody directed against the neuronal nuclei marker NeuN (BD Pharmingen) and a ^35^S-labeled Atf3, Bdnf, or calpastatin cRNA probes. Slices were first washed in sterile DEPC-treated 50 mM potassium phosphate-buffered saline (KPBS) and then incubated for 2 h at room temperature with NeuN (1:1000) antibody (Millipore) diluted in sterile KPBS plus 0.2% Triton X-100, 0.5% BSA (fraction V; Sigma-Aldrich) and 0.25% heparin sodium salt USP. Brain slices were then rinsed in sterile KPBS and appropriately incubated with a solution of Triton X-100, 0.5% BSA, 0.25% heparin sodium salt and biotinylated goat anti-rat IgG (1:500) or biotinylated goat anti-rabbit (1:1000) secondary antibody at room temperature for 2 h. Sections were then rinsed with KPBS and incubated at room temperature for 60 min with an avidin-biotin-peroxidase complex (Vectastin ABC Elite kit; Vector Laboratories), after which they were washed again in sterile KPBS. A solution of chromogen 3,3′-diamino-benzidine tetrahydrochloride (0.05%) and 0.003% H_2_O_2_ diluted in KPBS was applied to the brain slices and the ensuing peroxidase reaction was allowed to proceed for 10 min. Sections were then rinsed in KPBS, mounted onto Fisherbrand Colorfrost microscope slides, and underwent the *in situ* hybridization histochemistry described above using Atf3, Bdnf, or calpastatin ^35^S-labeled cRNA probe. Ethanol-dehydrating steps were shortened to 3–4 dips to minimize IHC signal loss.

**Table 1:** **Plasmids and enzymes used for the synthesis of the cRNA probes**.

**Plasmid**	**Vector**	**Lenght (bp)**	**Enzymes used for antisense probe**	**Source**
Mouse *Atf3*	PCRII-Topo	1430	Xbal/Sp6	Cloning: 5′ Primer ctg ggt cac tgg tat ttg agg att; 3′ aca aaa gtg cca tta aca tcc cac
Mouse *Bdnf*	PCRII-Topo	684	Spel T7	Dr. Steve Lacroix, Laval University; Cloning: 5′ Primer tac ctt cct gca tct gtt gg; 3′ cag cct tcc ttg gtg taa cc
Mouse Cast (calpastatin)	PCRII-Topo	1730	Xbal/Sp6	Cloning: 5′ Primer gcg gag caa gtc agg gtt gtc; 3′ atg tt gcc gga ctg
Mouse *Fox03a*	PCRII-Topo	839	Xhol/Sp6	Cloning: 5′ Primer ggc acc atg aat ctg aat gat g; 3′ acc aac aac gtt ctg tgt gga g
Mouse II*1a*	PCR blunt II	1985	Xhol/Sp6	Dr. P. W. Gray, Genetech Inc. San Francisco, CA, USA (Lomedico et al., 1984)
Mouse II*lb*	PCR blunt II	1360	Kpn1/T7	Dr. P. W. Gray, Genetech Inc. San Francisco, CA, USA (Gray et al., 1986)

### *In situ* hybridization analyses

*In situ* hydridization signals were measured on Biomax MR x-ray films (Kodak, Rochester, NY) using a Northern Light desktop illuminator (Imaging Research, Ste-Catherine's, Ontario, Canada) and a Sony (Tokyo, Japan) camera video system attached to a MicroNikkor 55 mm Vivitar extension tube set for a Nikon (Montreal, Quebec, Canada) lens and coupled to a Dimension GX270 personal computer (Dell Computer, North York, Ontario, Canada) and ImageJ software (version 1.23; W. Rasband, National Institute of Health, Bethesda, MD). The optical density (O.D.) for each pixel was calculated using a known standard of intensity and distance measurements from a logarithmic specter adapted from BioImage Visage 110s (Millipore, Ann Arbor, MI). Analyses for *in situ* hybridization signals were performed in the deeper half of the dorsolateral cortex, bilaterally, as illustrated in Figure 2C. Data for each mice consisted in the average O.D. signals from two separate slices located between bregma −0.82 mm and −1.58 mm on the anterior-posterior axis, and data for one slice consisted in the average O.D. of the values of the right and left hemispheres. Each value for a hemisphere was corrected for background signal by subtracting from it the O.D. value taken at a brain area devoid of positive signal.

### Fluoro-jade B staining

Every 12th section was mounted on Colorfrost/Plus microscope slides (Fisher Scientific, Pittsburgh, PA). The Fluoro-Jade B (FJB) staining procedure was employed to reveal neuronal death as described previously (Turrin and Rivest, 2006). Briefly, dried mounted brain sections were first fixed with 4% PFA vapors in a closed container placed in a water bath heated at 37°C for 2.5 h. The 4% PFA, in liquid form, was only present at the bottom of the container to not have the slides submerged. Slides were then let to air-dry and were dehydrated and rehydrated through graded concentrations of alcohol (50, 70, 100, 70, and 50% EtOH, 1 min each), and rinsed for 1 min in distilled water. Slides were then treated with potassium permanganate (0.06%) for 10 min and rinsed for 1 min in distilled water, followed by 0.004% FJB (Histochem, Jefferson, AR) in 0.1% acetic acid and 0.0002% 4′,6-diamidino-2-phenylindole (DAPI; Invitrogen, Burlington, ON, Canada) for 20 min. Slides were thereafter rinsed in distilled water (3 × 1 min), dried overnight at 37°C, dipped in xylene (3 × 2 min), and then coverslipped with distrene plasticizer xylene (DPX) mounting media (Electron Microscopy Sciences, Washington, PA).

### Fluoro-jade B quantification

Visualization of FJB-positive regions from the slides was done by using a C-80 Nikon microscope and super-high-pressure mercury lamp (Nikon) fitted with a Retiga EXi Fast digital camera (QImaging, Burnaby, BC, Canada) connected to a Precision 660 workstation (Dell Computer). As illustrated in Figure 2C, the bilateral deeper halves of the dorsolateral cortex were traced on a Wacom pen tablet (Vancouver, WA) by using the Neurolucida stereological software package (version 6.02.1; MicroBrightField, Williston, VT). The FJB-positive cells were then counted in the deeper half of the dorsolateral cortex, bilaterally, over two separate slices located between bregma −0.82 mm and −1.58 mm on the anterior-posterior axis. Data for each mice consisted in the average density of FJB-positive cell over the two slices.

### Statistical analyses

Data are expressed as mean ± standard error. Statistical analyses were performed with Two-Way ANOVA followed by Bonferroni *post hoc* test using Graph Pad Prism v5.0 software. Alpha level was set at 0.05.

## Results

### Kainic acid injection causes *De novo* synthesis of IL-1alpha and IL-1beta transcripts in the brain

Within 1 h after an intrastriatal injection of KA, mice developed significant convulsions, consisting in tremors, head-noddings and loss of balance. Mice displayed great variability however in the frequency and intensity with which they exhibited these signs, ranging from very mild to very strong displays of convulsive behaviors. In most cases, these signs largely disappeared by the start of the second day post-injection (i.e., after 24–36 h), although some still displayed subtle convulsive behaviors until the end of the third day. Finally, none of the mice that received the vehicle injection demonstrated any signs of convulsion.

**Figure 1:**
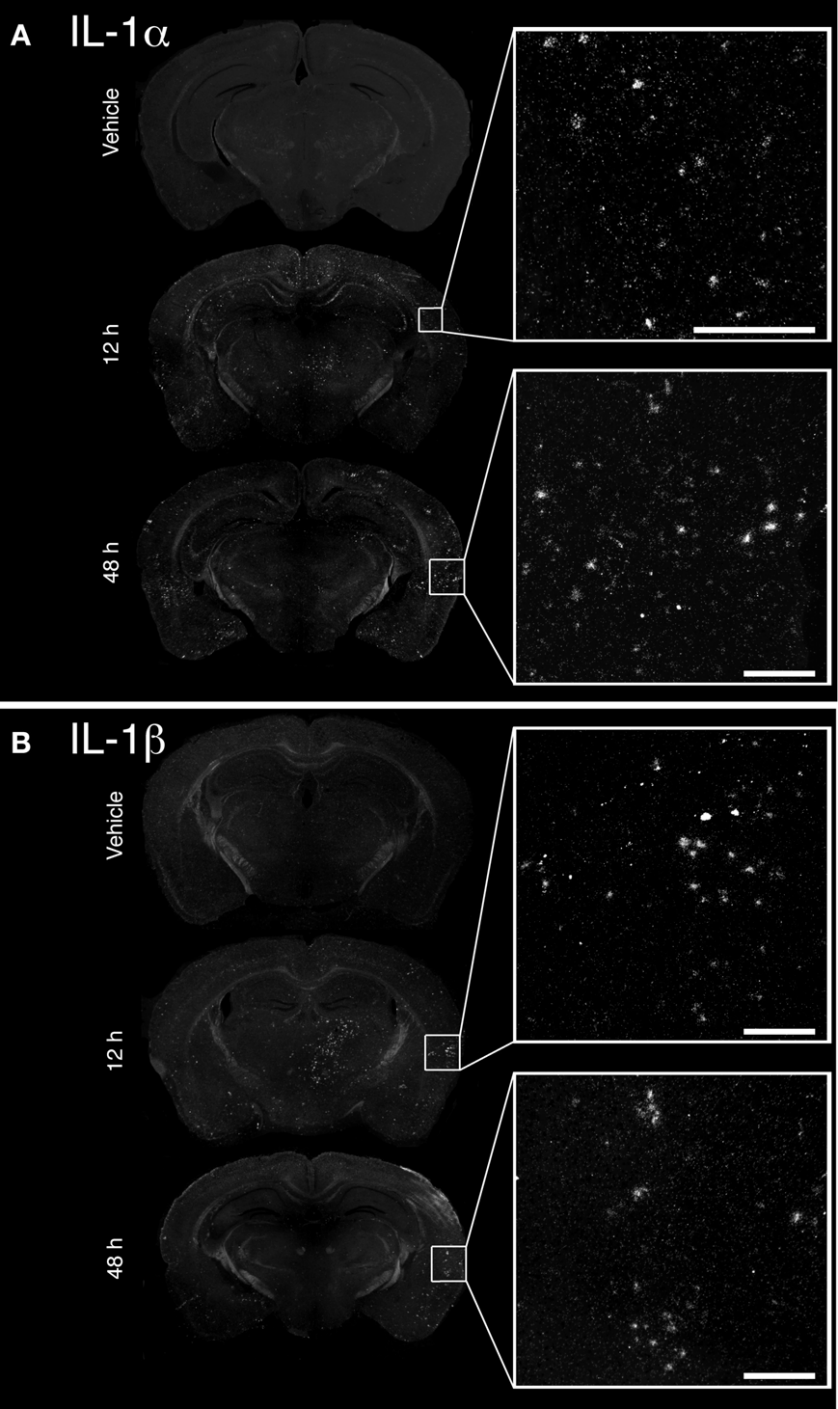
**Unilateral intrastriatal injection of kainic acid (KA) induces transcription of pro-inflammatory cytokines interleukin 1 alpha (IL-1α) and beta (IL-1β) throughout the brain.** (**A** and **B**) As depicted by dark-field photomicrographs, excitotoxicity triggered by intrastriatal injection of KA causes a strong induction of **(A)** IL-1α and **(B)** IL-1β gene transcription. mRNAs for these cytokines are clearly detected in numerous brain regions, including cortical and thalamic areas, at 12 and 48 h post-injection. No mRNA upregulation for these cytokines was detected in mice that received vehicle solution. Scale bars: 200 μm.

The convulsive behaviors of mice that received KA suggested that KA induced a strong dysregulation of neuronal activity, likely implicating excitotoxicity. As a large body of evidence suggests that KA-induced excitotoxicity is accompanied by an increase in the expression of numerous pro-inflammatory cytokines, we sought to confirm that cytokines IL-1α and IL-1β are *de novo* synthesized. Increased expression of IL-1α mRNA was detected at 12 and 48 h post-injection in the brain of mice that received KA, and was particularly strong in the cortex compared to other brain areas (Figure 1A). A clear upregulation of IL-1β mRNA at 12 h post-injection was also detected throughout the brain, including cortical and thalamic brain regions (Figure 1B). In contrast to IL-1α, IL-1β mRNA expression decreased dramatically at 48 h post-injection, suggesting that the expression of these cytokines is regulated by different mechanisms. Finally, expression for either cytokines remained undetectable in mice injected with vehicle. Overall, these data confirm that an intrastriatal injection of KA causes a strong induction of IL-1α and IL-1β cytokines in the brain, albeit with different kinetics. This suggests that these cytokines may play a role in modulating and coordinating the neuronal and inflammatory activities that are associated with KA-mediated excitotoxic insults.

### Excitotoxicity modulates similarly gene expression of transcription factors Atf3 and FoxO3a in WT and AcPb-deficient mice

**Figure 2:**
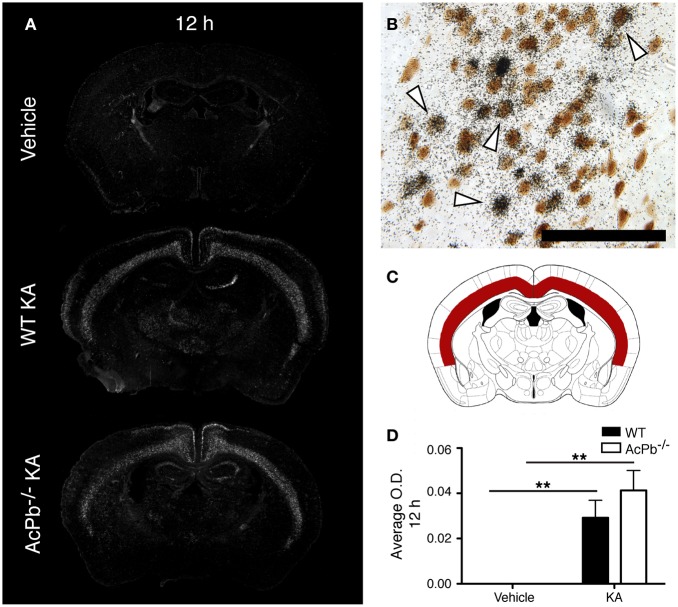
**IL-1RAcPb signaling does not modulate induction of Atf3 transcription factor mRNA in neurons following kainic acid (KA) injection.** (**A** and **D**) Intrastriatal injection of KA, but not vehicle injection, triggered bilaterally a profound increase in Atf3 mRNA in the deep layers of the dorsolateral cortex 12 h post-injection. This increase was similar in WT and AcPb-deficient mice. **(A)** Representative dark-field photomicrographs of dipped NTB2 emulsion. **(B)** Combination of *in situ* hybridization targeting Atf3 mRNA with immunohistochemistry (IHC) using NeuN antisera to label neurons revealed that Atf3 mRNA is upregulated in neurons. *White arrowhead*, positive co-localization. Scale bars: 100 μm. **(C)** Differential optical density analyses for Atf3 *in situ* hybridization signals were performed in the deeper half of the dorsolateral cortex, bilaterally. Note that all subsequent analyses were conducted in these same areas. **(D)** Optical density (O.D., in arbitrary units) of hybridization signal in the deep layers of the dorsolateral cortex 12 h post-injection, as described in “Materials and Methods.” Data are means ± SEM (bars) values from 3 to 8 mice per group. Significant differences were established by Two-Way ANOVA followed by Bonferroni *post hoc* test. ^**^p < 0.01.

Multiple mechanisms are activated in neurons to help them cope with the metabolic and oxidative stresses that develop following their dysregulated and excessive firings triggered by KA. Previous research showed that the activity of activating transcription factor 3 (Atf3) confers neuroprotective effects in contexts of calcium-mediated excitotoxicity (Francis et al., 2004; Zhang et al., 2009, 2011). As depicted by the Figure 2A, a strong induction of Atf3 mRNA is detected in the brain of mice that received KA, but not in mice that received vehicle. However, the spatial pattern of Atf3 expression was quite variable among the mice, possibly reflecting intrinsic differences in vulnerability and susceptibility to KA-induced excitotoxicity. Atf3 induction was strongest at 12 h post-injection, and returned to basal levels by 48 h (data not shown), which paralleled the temporal pattern of convulsive behaviors. Furthermore, colocalization experiments with neuronal maker NeuN indicated that Atf3 mRNA upregulation occurred exclusively in neurons (Figure 2B). Interestingly, the initial study by Smith et al. (2009) that reported the discovery of AcPb also provided evidence that this adaptor protein can modulate transcription of Atf3 in response to IL-1β (Smith et al., 2009). Thus, we tested the hypothesis that the increased transcription of the Atf3 gene is regulated by signaling effectors downstream of AcPb. To test this hypothesis, we measured by optical densitometry Atf3 transcript levels within the deeper half of the cerebral cortex, bilaterally (Figure 2C). This brain region was selected because it showed a consistent pattern of expression across all the mice that received KA. However, no significant differences in Atf3 mRNA expression between WT and AcPb^−/−^ mice were noted at 12 h post injection (Figure 2D). This result suggests that in a complex *in vivo* setting, AcPb signaling has little, if any, impact on the induction of transcription of the *Atf3* gene.

**Figure 3:**
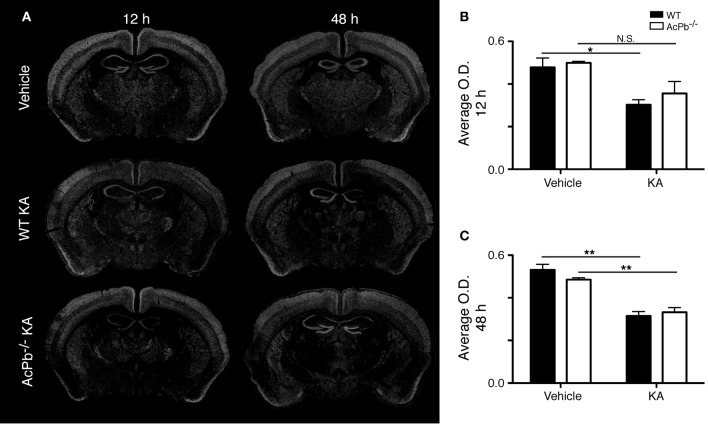
**Decrease of FoxO3a mRNA in the deep layers of the dorsolateral cortex in WT and AcPb-deficient mice following intrastriatal injection of kainic acid (KA). (A)** Dark-field photomicrographs of dipped NTB2 emulsion slides showing expression of Foxo3a mRNA at 12 and 48 h post-injection of KA. (**B** and **C**) Differential optical density (O.D., in arbitrary units) of hybridization signal in the bilateral deep layers of the dorsolateral cortex at **(B)** 12 and **(C)** 48 h post-injection, as described in “Materials and Methods.” Data are means ± SEM (bars) values from 3 to 5 mice per group. Significant differences were established by Two-Way ANOVA followed by Bonferroni *post hoc* test. ^*^p < 0.05, ^**^p < 0.01.

Another signaling system implicated in neuroprotection during excitotoxicity involves the transcription factor forkhead box O3a (FoxO3a) (Mojsilovic-Petrovic et al., 2009; Dick and Bading, 2010). We thus investigated whether its expression is modulated during excitotoxicity. Interestingly, within the same areas of the cortex that exhibited a strong Atf3 mRNA induction, we found a decrease in the expression of the FoxO3a transcript at both 12 and 48 h post-injection (Figures 3A,B, and C). However, this decrease was essentially similar in WT and AcPb^−/−^ mice. Thus, FoxO3a is not modulated at the transcriptional level by AcPb signaling.

Overall, these results suggest that in a context of excitotoxicity, signaling downstream of AcPb does not regulate the mechanisms implicated in the expression of transcription factors Atf3 and FoxO3a that are relevant to the modulation of neuronal survival.

### No significant difference in Bdnf mRNA transcription in WT compared to AcPb^−/−^ mice

**Figure 4:**
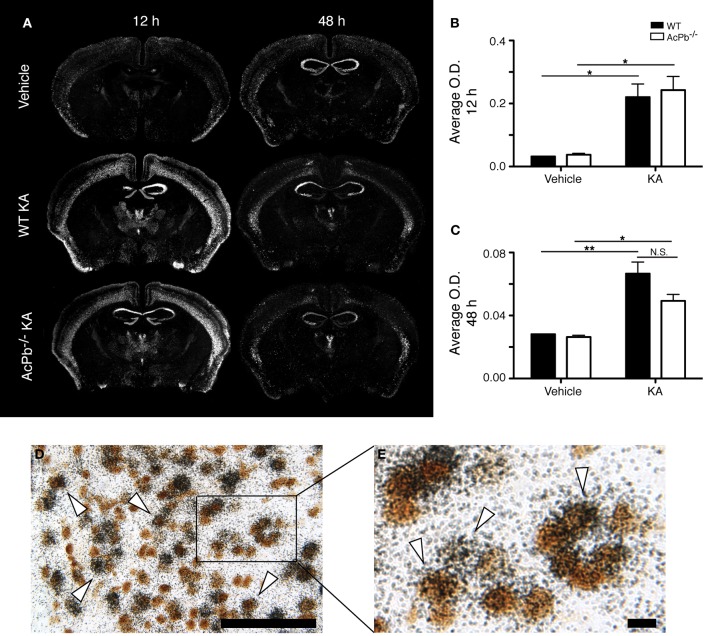
**Similar induction of neuronal Bdnf mRNA expression in WT and AcPb-deficient mice in response to excitotoxicity triggered by an intrastriatal injection of kainic acid (KA). (A)** Representative dark-field photomicrographs of dipped NTB2 emulsion slides showing expression of Bdnf mRNA at 12 and 48 h post-injection of KA. (**B** and **C**) Differential optical density (O.D., in arbitrary units) of hybridization signal in the bilateral deep layers of the dorsolateral cortex at **(B)** 12 and **(C)** 48 h post-injection, as described in “Materials and Methods.” Data are means ± SEM (bars) values from 3 to 8 mice per group. Significant differences were established by Two-Way ANOVA followed by Bonferroni *post hoc* test. (**D** and **E**) Neuronal expression of Bdnf mRNA was confirmed by combining *in situ* hybridization with immunohistochemistry using antisera directed against NeuN. *White arrowhead*, positive co-localization. Scale bars: **(D)** 100 μm, **(E)** 10 μm. ^*^p < 0.05, ^**^p < 0.01.

An intimate relationship exists between synaptic activity and neurotrophin brain-derived neurotrophic factor (Bdnf), in which both mutually regulate the activity of each other (Poo, 2001; Greenberg et al., 2009; Pozo and Goda, 2010). Indeed, Ca^2+^-dependent mechanisms modulate positively the release of Bdnf, which in turn modulates signaling pathways involved in synaptic plasticity (Pozo and Goda, 2010). Bdnf signaling has frequently been associated with neuroprotection following excitotoxic insults (Glazner and Mattson, 2000; Gratacos et al., 2001; Almeida et al., 2005; Gobbo and O'Mara, 2005; Jiang et al., 2005; Bemelmans et al., 2006; Bovolenta et al., 2010; Vidaurre et al., 2012). Also of interest, IL-1β may interfere with Bdnf signaling in neurons (Tong et al., 2008). Thus, we investigated the possibility that IL-1 signaling through AcPb modulates Bdnf mRNA transcription by assessing the expression level of the Bdnf transcript in the deeper half of the cortex, bilaterally. KA injection caused a very strong increase in the transcription of the *Bdnf* gene in cortical neurons at 12 h post-injection, and this increase was similar in WT and AcPb-deficient mice (Figures 4A,B,D, and E). At 48 h, Bdnf mRNA levels decreased compared to 12 h, but were still elevated compared to vehicle-injected mice (Figure 4C). Note also that to the extent that Bdnf mRNA synthesis is indicative of synaptic activity, these data suggest that the initial early response to KA is similar in WT and AcPb-deficient mice.

### AcPb signaling promotes a delayed transcription of calpastatin during excitotoxic insults

**Figure 5:**
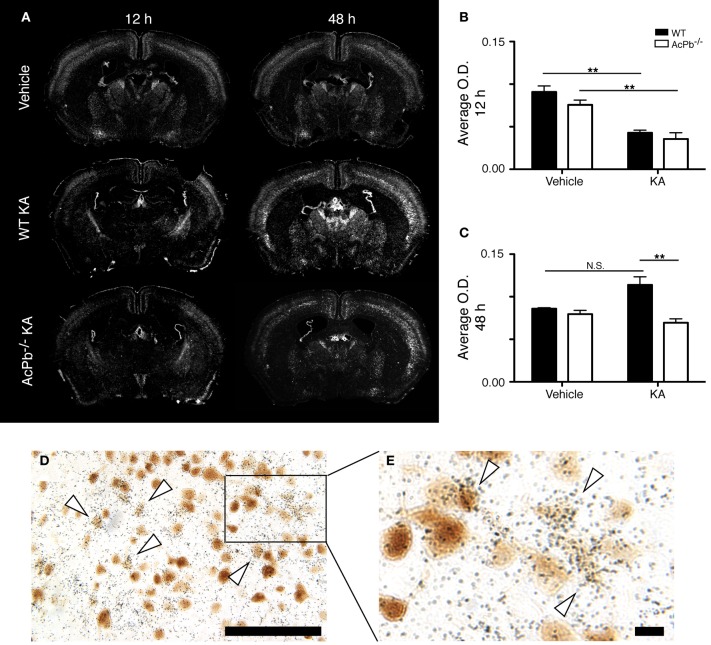
**AcPb signaling is necessary for the delayed increase of transcription of the calpastatin gene in cortical neurons following intrastriatal injection of kainic acid (KA). (A)** Representative dark-field photomicrographs of dipped NTB2 emulsion slides showing bi-phasic expression of calpastatin mRNA at 12 and 48 h post-injection of KA. (**B** and **C**) Differential optical density (O.D., in arbitrary units) of hybridization signal in the bilateral deep layers of the dorsolateral cortex at **(B)** 12 and **(C)** 48 h post-injection, as described in “Materials and Methods.” Data are means ± SEM (bars) values from 3 to 8 mice per group. Significant differences were established by Two-Way ANOVA followed by Bonferroni *post hoc* test. (**D** and **E**) Neuronal expression of calpastatin mRNA was confirmed by combining *in situ* hybridization with immunohistochemistry using antisera directed against NeuN. *White arrowhead*, positive co-localization. Scale bars: **(D)** 100 μm, **(E)** 10 μm. ^**^p < 0.01.

Numerous lines of evidence suggest a significant implication of the calpain-calpastatin system in neuronal excitotoxic pathologies (Wu et al., 2004; Higuchi et al., 2005, 2012; Takano et al., 2005; Araujo et al., 2007, 2008; Cao et al., 2007). Calpains are cysteine proteases and are important effectors of neuronal damage during ischemia, oxygen-glucose deprivation, and NMDA-induced neuronal death (Cao et al., 2007; Bevers et al., 2009, 2010). Importantly however, the activity of neuronal calpains is inhibited by calpastatin, and this protein confers significant neuroprotection in models implicating excitotoxicity (Higuchi et al., 2005; Takano et al., 2005). We thus examined whether KA-induced excitotoxicity is associated with changes in calpastatin gene transcription, and whether those changes are modulated by AcPb signaling. As can be seen on Figure 5, a bi-phasic modulation of calpastatin mRNA expression level occurred in cortical neurons following KA injection. Indeed, 12 h following KA injection, calpastatin mRNA levels decreased similarly in WT and AcPb^−/−^ mice (Figures 5A and B). However, at 48 h post-injection, calpastatin mRNA levels recovered (Figures 5A and C). At that time, WT mice that received KA actually displayed a strong trend towards an increased expression compared to vehicle-treated mice, although this effect was not statistically significant (Figures 5A and C). Interestingly, this recovery and possible enhancement are dependent on signaling events downstream of AcPb, as calpastatin mRNA expression at 48 h in AcPb-deficient mice was significantly lower than that of WT. Calpastatin mRNA levels in AcPb^−/−^ mice remained 17% lower than those of mice that received vehicle at 48 h. These results therefore suggest that AcPb signaling provides an important positive regulatory input in the modulation of a key mechanism of neuroprotection against KA-induced excitotoxicity.

### AcPb signaling during excitotoxic insults promotes long-term neuronal survival.

**Figure 6:**
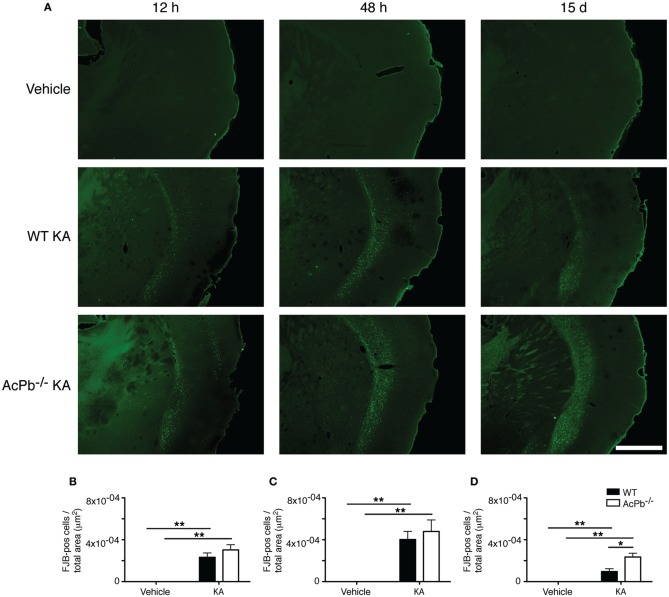
**AcPb promotes long-term survival of cortical neurons following induction of excitotoxicity by intrastriatal injection of kainic acid (KA). (A)** Representative photomicrographs of fluoro-jade B (FJB) staining revealing neuronal death at 12 and 48 h, and 15 days post-injection of KA. **(B–D)**. Stereological quantification of FJB-positive cells in the bilateral deep layers of the dorsolateral cortex at **(B)** 12 h, **(C)** 48 h, and 15 days post-injection **(D)**. Data are means ± SEM (bars) values from 3 to 8 mice per group. Significant differences were established by Two-Way ANOVA followed by Bonferroni *post hoc* test. Scale bar: 1.0 mm. ^*^p < 0.05, ^**^p < 0.01.

The significant increase in calpastatin mRNA expression in WT compared to AcPb^−/−^ mice, combined with the strong trends toward higher levels of Bdnf transcripts in WT mice at 48 h, suggest that signaling events downstream of AcPb could confer neurons with significant protection against calcium-related disease mechanisms and thus promote neuronal survival in a context of excitotoxicity. We examined this possibility by quantifying neuronal damage marker FJB-positive signal/cells in the deeper half of the cerebral cortex, bilaterally. Injection of KA, but not vehicle, led to significant neuronal damage, as depicted by the presence of numerous FJB-positive cells (Figure 6). Extensive damage already had occurred by 12 h post-injection and it further increased at 48 h. However, quantification of FJB cells did not reveal any differences between WT and AcPb^−/−^ mice at either 12 or 48 h (Figures 6A,B and C). Although this was not consistent with our initial prediction, we reasoned that these time points may have been too early to assess the possible beneficial effects of the increased expression of calpastatin that had just begun to proceed. To address this issue, we conducted another experiment in which mice were allowed to survive for 15 days after the injection of KA. Interestingly, WT mice at that time displayed significantly less FJB-positive cells in the deeper half of the cerebral cortex than AcPb^−/−^ mice (Figures 6A and D). This suggests that AcPb signaling regulates adaptive mechanisms in neurons that contribute substantially to their long-term protection following excitotoxic insults.

## Discussion

A wide range of different types of excitotoxic lesions and excessive dysregulations of calcium homeostasis are intrinsically linked to neurodegenerative diseases. In response to these insults, IL-1 cytokines are secreted to stimulate the activity of the innate immune system and to adapt the cellular activity of neighboring cells to prevent spread of damage and to eventually promote brain repair. We provide here evidence that a neuron-specific IL-1 signaling pathway downstream of adaptor protein IL-1RAcPb may enhance the ability of cortical neurons to cope and survive excessive calcium influx and/or the strong oxidative stress that accompanies an acute inflammatory response. Indeed, signaling through AcPb in neurons promoted a delayed transcription of calpastatin, which in turn could presumably inhibit the pro-death activities of calpains. This observation not only confirms the neuroprotective properties of AcPb signaling, as originally reported by Smith et al. (2009), but it also expands upon it by identifying a potential mechanism accounting for its neuroprotective effects. This study also further highlights the intimate link that exists between AcPb and the regulation of calcium signaling in neurons recently suggested by Huang and colleagues (2011).

The inhibitory properties of calpastatin on the activity of the calpain proteases are very potent. Calpastatin has a very high binding affinity for both μ- and m-calpains in the presence of calcium, and one calpastatin molecule can inhibit up to four calpain molecules (Maki et al., 1988). Many studies have revealed a major neuroprotective role for calpastatin in a variety of brain lesions involving excitotoxicity. For example, calpastatin deficiency in mice increased neuronal death following intra-hippocampal injections of KA (Takano et al., 2005). In contrast, neurons of transgenic mice overexpressing calpastatin exhibited an enhanced resistance to excitotoxicity-induced death (Higuchi et al., 2005; Takano et al., 2005). Furthermore, a study recently reported that calpastatin is neuroprotective in a model of excitotoxic-related apoptosis, but not during excitotoxicity-related necrosis (D'Orsi et al., 2012). Our data seem to be in agreement with this latter observation, as we found a protective effect for AcPb signaling at 15 days post-injection, but not at 12 or 48 h. Note however that the exact mechanism by which AcPb signaling promotes transcription of calpastatin remains to be elucidated. Evidence suggests that the activation of Src kinases by AcPb, as reported by Huang et al. (2011), could be a key factor. Indeed, Src in neurons can regulate the PI3K-Akt signaling pathway, which in turn controls cAMP response element-binding protein (CREB) (Crossthwaite et al., 2004). Interestingly, CREB promotes transcription of the calpastatin gene (Arroba et al., 2009).

The present study suggests that there is a context-specificity within the spectrum of excitotoxic lesions in which AcPb signaling confers neuroprotection. Although no electrophysiological analyses were performed, we found no evidence that lack of AcPb abrogated the initial response of neurons to KA in mice, as expression of Atf3, FoxO3a, Bdnf, and calpastatin mRNAs at 12 h, and FJB counts at 12 and 48 h, were similar in WT and AcPb^−/−^ mice. Note that it is possible that the high concentration of KA in the cerebral milieu shortly after the injection may have masked the effects of a regulatory role of AcPb on neuronal activity at the early time point. However, over time the excitotoxic insults evolve and the sustained stimulation of neuronal circuitries necessarily becomes more dependent on endogenous, more physiological concentrations of glutamate and Ca^2+^. As this occurs, reorganization of intracellular signaling and metabolic pathways may proceed to allow surviving neurons to deal with prolonged Ca^2+^, metabolic, and oxidative stresses. This is the pathophysiological context in which AcPb signaling may contribute significantly to neuroprotection. Indeed, AcPb could provide substantial regulatory activity on the establishment of a neuroprotective, calpastatin-dependent mechanism that increases the resistance of neurons to chronic, high glutaminergic excitations and sustained elevated influx of Ca^2+^. Our results showing that a higher, delayed expression of calpastatin mRNA in WT mice compared to AcPb-deficient mice 48 h after KA injection is accompanied by a lower level of neuronal damage, as measured by FJB at day 15 post-injection, support this possibility. Importantly, these results are consistent with a study by Stifanese et al. (2010) that also provided evidence that adaptive changes within the calpain-calpastatin system, including an induction of calpastatin transcription, might be key to allow neurons to cope with prolonged elevated intracellular levels Ca^2+^ (Stifanese et al., 2010). Note however that the magnitude of the excitotoxic lesion may be a determining factor as to whether upregulation of calpastatin gene transcription through endogenous signaling pathways can proceed and provide a neuroprotective effect, as we found no evidence that this gene is modulated in the brain of APP^PS1/SWE^ mice despite the presence of senile plaques (Laflamme, Rivest, unpublished observation).

On a more general note, the present study is among the first to provide *in vivo* evidence for an intrinsic neuronal protective mechanism modulated by IL-1 signaling. Indeed, most studies investigating the effects of IL-1 cytokines initiated as a consequence of excitotoxic insults have typically observed a detrimental outcome on neuronal survival for IL-1 signaling. It is worth noting however that those studies that investigated the role of IL-1 cytokines in such settings have used experimental paradigms involving the complete inhibition of their activities, either by injecting recombinant IL-1RA, or using mice deficient for the different components of IL-1 signaling (i.e., Relton and Rothwell, 1992; Vezzani et al., 2000). Such paradigms could hardly dissociate the effects of IL-1 signaling in promoting the acute inflammatory response from those more specifically involved in the regulation of signaling pathways that allow neurons to cope with excessive glutaminergic and calcium stimulations. In fact, the role of IL-1 cytokines in modulating cellular responses in a cell-specific manner, *in vivo*, has actually received little attention to this day, and significant neuroprotective mechanisms may thus have been overshadowed. In addition, our results also suggest that it may be valuable to precisely examine the role of IL-1 signaling during excitotoxic insults in a cell-type specific manner by using conditional knockout mice models. This strategy could prove to be quite helpful in determining whether the overall net effect of IL-1 signaling is typically associated with a detrimental outcome for neurons because it promotes an overwhelming inflammatory response or because it negatively affects the intrinsic ability of neurons to adapt and survive excitotoxicity-related damage.

In sum, this study suggests that during acute excitotoxic insults, IL-1RAcPb signaling in neurons promotes a delayed transcription of calpastatin, which in turn could provide substantial protection to neurons from the long-term damaging and toxic effects of excessive calcium stimulations. It will be critical to determine in the future whether such adaptive mechanism also plays a key role in the etiology and progression of more chronic neurodegenerative diseases.

### Conflict of interest statement

The authors declare that the research was conducted in the absence of any commercial or financial relationships that could be construed as a potential conflict of interest.
